# A critical review of research into mental health consumers' perspectives on their physical health: Is there an absence of consumers in the design, conduct, analysis and reporting of this research?

**DOI:** 10.3389/fpubh.2022.982339

**Published:** 2023-02-06

**Authors:** Chloe R. Green, Rosiel Elwyn, Nicholas Hill, Kate Johnston-Ataata, Renata Kokanović, Chris Maylea, Grace McLoughlan, Russell Roberts, Stuart D. M. Thomas

**Affiliations:** ^1^School of Law, La Trobe University, Melbourne, VIC, Australia; ^2^Psychology and Social Sciences, University of the Sunshine Coast, Maroochydore, QLD, Australia; ^3^School of Social and Political Sciences, The University of Melbourne, Parkville, VIC, Australia; ^4^School of Global, Urban and Social Studies, RMIT University, Melbourne, VIC, Australia; ^5^School of Business, Charles Sturt University, Bathurst, NSW, Australia

**Keywords:** mental health, physical health, consumer, co-production, mental health service user

## Abstract

We conducted a critical review, using systematic methods, of the literature examining mental health consumer perspectives on their physical and mental health in academic research published between 2005 and 2021. This review examined the inclusion, extent, type and centrality of consumer perspectives regarding their mental and physical health. The search produced 1,865 papers from which 116 met the inclusion criteria. Studies predominantly focused on consumers' individual experiences of their physical and mental health, including but not limited to their understandings and experiences of medication and associated risk factors. They also captured some social aspects of mental health consumers' physical health, including factors that impacted individual agency, stigma, and social and interpersonal factors. Structural factors affecting physical and mental health, such as accessibility of services and financial constraints, were also identified. The review revealed that in comparison to clinician perspectives, the direct representation of consumer perspectives was lacking. Similarly, while clinician and carer perspectives on structural factors were investigated, the consumer perspective in this area was missing. The review also found few genuine codesigned or coproduced research studies. To better identify and respond to the health needs as prioritized by consumers, this paper argues it is imperative that future studies prioritize codesigned and coproduced research. It is argued that a focus on “services as provided” rather than “services as received” has contributed to a lack of progress in addressing the life expectancy gap for consumers. It is recommended that journals, ethics committees and research policy organizations develop guidelines and standards to inform best practice in research on consumer perspectives and experience and to support the implementation of codesigned and/or coproduced approaches in future research. Respecting and including consumers as equal partners in the research process will lead to more meaningful insights to inform policy and practice and reduce the life expectancy gap for people living with mental health concerns.

## 1. Introduction

As a group, people with a lived experience of mental health concerns have far worse health and life expectancy outcomes than the rest of the population (1). Mental health consumers experience two times the rate of early death compared to the general population, and in rural Australia, this rate is three times higher (2)^1^. While the last decade has seen an increasing emphasis placed on lived experience and consumer engagement in service design and planning (4), there remains a relative lack of consumer voices within published literature regarding their physical and mental health. A recent narrative literature review [(5), p. 37] concluded “there is a lack of consumer voices both in research findings and in the research production. This lack… may be one of the major reasons why so little progress has been made in addressing the health and mortality gap for people living with mental health concerns.” Furthermore, extant literature tends to over-emphasize individual health responsibilities at the expense of social factors that influence health status. The purpose of this literature review was to (1) distill consumer experiences, perspectives, and understandings concerning their physical and mental health and (2) ascertain the role of consumers in the design of research and across all aspects of the research production process.

## 2. Background

The early mortality of people with mental health concerns has been associated with increased risk for cardiovascular disease, cancer, and respiratory diseases (6, 7). In addition to increased premature mortality, consumers generally experience poorer physical health, increased incidence of chronic disease and decreased quality of life (8, 9). Medication side effects, diet, exercise and smoking have all been associated with health inequities (10), but many social factors also impact consumers. These include socioeconomic status, social participation or exclusion, stigma and health-related discrimination (11). For example, Australian consumers not in full-time employment are six times more likely to die prematurely than the general population (12).

These poor health outcomes should be understood as partially produced by the effects of stigma and structural discrimination in the healthcare system. For example, while mental health consumers have a high rate of death due to heart disease, medical procedures for improving blood flow to the heart are less common than for the general population, especially for persons experiencing psychosis (13–16). Furthermore, despite consumers' high risk for other cardiovascular illnesses, they are screened for high cholesterol and recommended treatments to lower cholesterol at lower rates than the general population (17–19). Finally, consumers experience lower rates of hospitalization for physical health concerns than the general population and lower incidences of treatment when hospitalized for their health concerns (13, 15, 19–22). These findings suggest structural factors and the social determinants of health (23) play an outsized role in determining the physical health outcomes of consumers.

Many consumers experience “diagnostic overshadowing,” where their physical health issues will be misattributed to their mental distress (24). A recent study indicates that only 1 in 5 mental health professionals asked about consumers' physical health (25). This may be due to a singular focus on the presenting problem, lack of time, a presumption that other health professionals will address social and physical issues or lack of training (26). Nash (27) argues that focusing on mental health over physical health and holistic care causes delays and gaps in treatment due to discrimination and stigma in healthcare encounters. Regardless of the cause, diagnostic overshadowing is a significant factor in the poor health and early death of people with mental health concerns. Consumers also frequently report feelings of dismissal when seeking healthcare. Recent research into consumers' experience of physical health care health professionals indicated that when consumers raised health concerns or medication side-effect concerns, only half of the mental health professionals took these concerns seriously (25). This is problematic because, as Happell et al. (28) notes, feeling dismissed can result in mental health consumers not reporting physical health events, potentially compounding adverse health impacts over the longer term. The frequency of such encounters in primary care, alongside an often-discontinuous experience of the mental health care system, directly contributes to poor health outcomes.

Authentic lived experience engagement in research can repair what some writers have referred to as the “silencing of consumer voices” and support a greater understanding of the experiences and needs of consumers, fostering a deeper understanding of the interconnections between people's mental and physical health (29, 30). Increasingly, policy and governmental agencies are investing in coproduction as a part of best practice in mental health. A recent report from the World Health Organization included a directive to empower and involve consumers in all aspects of mental health provision, research, and policy (31). Supporting this stance, the recent Royal Commission into Victoria's Mental Health System (32) highlighted the need for consumer collaboration as a central factor influencing the future direction of mental health services. Accordingly, this review sought to capture the inclusion, extent, type and centrality of consumer perspectives regarding their mental and physical health in the international literature.

## 3. Method

We conducted a critical review using systematic methods in six databases; CINAHL, Informit, Psychinfo, PubMed, Scopus and Web of Science.^2^ Peer-reviewed articles published in the English language between 2005 and 2021 were eligible to be included. In addition, we also conducted a gray literature search using the same search terms, for the maximum searchable results on Google Scholar, in addition to any unpublished material represented in some of the databases searched.^3^ We selected the cut-off date of 2005 based on the parameters of the earlier review (5). As this is not a systematic review, but a critical review using systematic methods, PRISMA was not used. To identify studies with a coproduced or codesigned methodology, we recorded studies that disclosed a prominent lived experience component in their methods (participatory action research, codesign, coproduced), or the inclusion of a dedicated lived experience researcher as part of the research team design.

### 3.1. Search strategy

Searches were conducted between May and June of 2021. Search terms were arrived at in consultation with an associated Consumer Leadership Advisory Group, led by a consumer researcher, who reviewed the terms proposed and suggested alternatives or additions where appropriate, such as “patient engagement.” These terms were applied to the title, abstract, and keywords (where possible). Keywords included:

“physical health” OR “physical illness” OR “life expectancy” OR “quality of life” OR “physical^*^ health” OR “physical disability” OR “comorbid” OR “co-morbid” OR “mortality” OR “cardiovascular” OR “diabetes” OR “metabolic,” AND “mental illness” OR “mental health” OR “schizophrenia” OR “schizo^*^” OR “bipolar,”
AND “qualitative” OR “consumer” OR “focus group” OR “patient^*^ perspective” OR “lived experience” OR “interview” OR “peer” OR “service user” OR “patient engagement.”

In consultation with our Consumer Leadership Advisory Group, these specific physical health diagnoses were included as they were identified in the extant literature as the major contributors to early morbidity for people with pre-existing mental health diagnoses. The specific mental health diagnoses were included as they are most linked to early morbidity and are not commonly identified in the literature as arising from physical health conditions. For example, many studies identified that people with a terminal diagnosis may subsequently develop depression, but that is not the focus of this review.

From this initial search, 1,845 articles were identified. Of these, 1,296 duplicates were removed, leaving 549 articles to be reviewed for potential inclusion. Studies were included if they (1) included consumer perspectives on mental and physical health and (2) were published between 2005 and 2021. Studies were excluded if they met one or more of the following criteria: (1) presented only clinician or carer perspectives, (2) were reviews of existing research, (3) focused on child or youth mental health, and (4) assessed or evaluated the implementation of programs (unless the source also included substantial consumer perspective on issues outside the program evaluation).

The search strategy purposefully excluded studies that were solely quantitative in design. Such research commonly surveys a large number of respondents and aggregates their responses. While this can effectively reveal trends, distributions and averages from the data, the research questions are framed by researchers and this method cannot directly represent individual consumer statements or viewpoints, so such studies were excluded.

**Figure 1:**
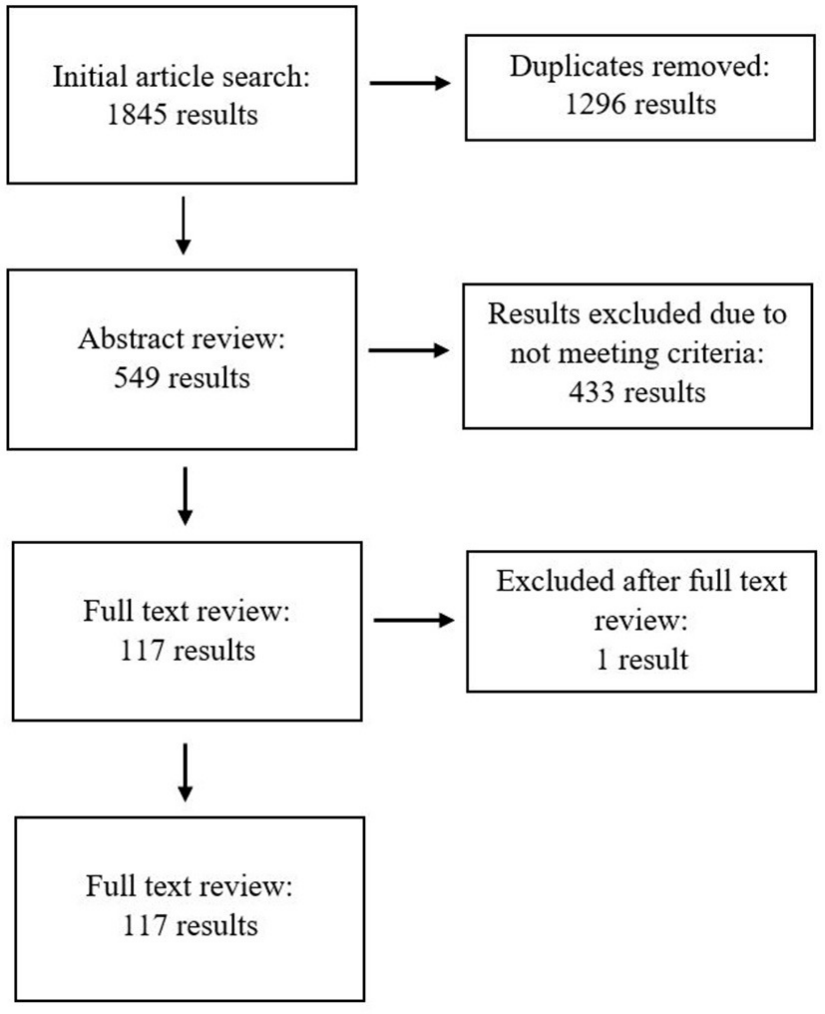
Flowchart of database searching methods.

Of the 549 articles that progressed to abstract review, 433 results were excluded due to not meeting one or more of the inclusion criteria above. Consequently, 117 results were included in the full-text review conducted by one of our researchers, with one article excluded during the full-text review. A total of 116 articles were thus reviewed and analyzed for their level of consumer perspective and their discussion of physical and mental health experiences (see Figure 1). The initial analysis was conducted by a team including lived experience and non-lived experience researchers, then cross-checked for scholarly rigor by additional team members.

### 3.2. Limitations

This review was confined to articles published between 2005 and 2021. These parameters were selected based on the relative nascency of consumer research and the consistency of terminology used to define the consumer movement within health research. From Roberts' (5) prior literature review, we found a sharp drop-off in relevant sources between 2005 and 2007, so we are confident that few relevant authorities would have been published before 2005. However, this review may have excluded some early research in this field.

Many articles are published on related physical and mental health topics, such as lifestyle-focused studies addressing fitness programs or smoking cessation, without linking these interventions to physical health conditions. Similarly, many studies examined the mental health consequences of poor physical health. In some cases, these topics are also featured in studies considered in this review; however, further research is required to examine the presence of consumer perspectives in these related fields.

## 4. Experience of and contributors to physical health for mental health consumers

The findings from our review are presented in two sections. In this first section, we discuss our findings as they relate to consumers' experience of physical and mental health and how contributing factors were understood. This section is divided into two subsections; individual and social aspects. We then identify in the following section key challenges and gaps within the literature before concluding the article with recommendations for future research.

### 4.1. Individual experiences of physical health and mental health

As discussed in Section 4.2, many consumers understood their physical health issues as relating to broader social and systemic issues. Despite this, the literature largely focused on consumers as individuals, identifying poor physical and mental health issues with individual choices or experiences. We begin by noting this framing, before summarizing how consumers placed value on good health, then discussing consumers' relationships to medication. We then examine how consumers conceive of and communicate their agency and expertise.

#### 4.1.1. Individualistic framing of physical health and mental health

The literature overwhelmingly frames physical and mental health as a challenge to be navigated by individual consumers rather than being understood within a social determinants of health framework. Studies generally focused on the category of “severe mental illness” rather than specific diagnoses; under a quarter (*n* = 27) focused on a specific mental health diagnosis—schizophrenia (*n* = 17), major depression (*n* = 4), bipolar disorder (*n* = 3), and generalized anxiety disorder (*n* = 3). Similarly, less than half (*n* = 40) considered specific physical health concerns; namely, diabetes (*n* = 12), cardiovascular health (*n* = 10), cancer (*n* = 2), metabolic syndrome (*n* = 2), chronic obstructive pulmonary disease or other respiratory diseases (*n* = 4), dental health (*n* = 2), HIV (*n* = 1), eating disorders (*n* = 1), and other unspecified chronic illnesses (*n* = 4). Of the remaining studies, just under half (*n* = 56) examined broad concepts of physical health, including exercise, diet, and smoking habits. These studies were primarily concerned with lifestyle changes consumers could make to improve their physical health, rather than addressing systemic factors contributing to poor physical health or specific health conditions, such as accessibility of services and financial constraints. This focus on individual-related factors implies an emphasis on the individual as a site for change or intervention and limited consideration of social and systemic factors that may contribute to poor health outcomes.

#### 4.1.2. Why good physical health is important

Consumers felt their physical and mental health were interconnected and part of a holistic system of health (35–37). Physical health was identified as having the potential to negatively impact mental health, and vice versa; consumers understood this interrelationship as affecting their quality of life (35, 38). Accordingly, good physical health was seen as critical to mitigating the negative symptoms of mental health concerns (39, 40). Consumers also reported having to divide mental, financial and emotional resources between their physical and mental health (40–42). In other studies, consumers noted that access to group physical activity had the additional benefit of mitigating social isolation stemming from their experiences of mental health challenges, but also suggested that good physical health may be a prerequisite to social and community participation (39, 43, 44).

#### 4.1.3. Understandings of medication and side effects

The use of neuroleptic or psychotropic medication is known to be a significant factor negatively impacting the physical health of consumers. Medication and side effects featured in many papers (*n* = 102), with 26 papers exploring consumers' experiences of mental health medication and their effects on physical health. The impact of psychotropic medications on an individual's physical health was commonly cited, with side effects including increased appetite, weight gain, and tremors or excessive sweating (36, 45–49). Whilst some studies focused primarily on the impact of weight gain from neuroleptic use on body image, other studies showed some consumers were making connections between medication use and broader health risks [see, e.g. (41)].

This review also identified intersecting viewpoints around the challenges of taking such medications, in terms of agency, conflicting health priorities, and medication burden, as well as the social and emotional strains of sustaining what can often be a complex medication routine. Consumers felt that their concerns about medication side effects were not taken seriously by psychiatrists, or expressed a general reluctance to raise concerns in the first place, leading them to become resigned to the physical impacts of their medication (42, 50). A common theme was that consumers felt the use of psychotropic medication had a direct negative impact on their general physical health and wellbeing, as well as their ability to effectively manage co-occurring chronic physical illness (41, 42, 51, 52). The side effects of mental health medications were cited as a common factor driving the choice to stop taking medication (53) or avoid primary care providers (54). These findings indicate a breakdown in the therapeutic relationship and a failure in collaborative care. Medication burden was identified as a significant challenge for consumers who were managing medications and multiple side effects, as well as the financial costs associated with treatment (55). Such positions should prompt future research to consider not just the clinical side effects but also how people navigate the range of factors that inform decisions around medication use.

#### 4.1.4. Individual agency and expertise

Several key themes were apparent in the published literature relating to emotional and relational dimensions of physical and mental health concerns. Motivation was discussed in 17 articles and was framed by some researchers as deficiency of motivation negatively affecting health, and by others as a positive force to implement lifestyle changes. Several articles looked at lack of motivation as a reason why consumers may not enact recommended lifestyle changes (36, 43, 45, 56) or a driver of continued smoking habits (57). Others investigated the positive impacts of peer support as a motivator to increased physical exercise (58), what types of physical activities consumers would be motivated to take part in Graham et al. (59), and whether motivational or behavioral interventions improved physical health outcomes (60). Studies tended to frame the concept of motivation as a quality residing in the individual (self-motivation driving or impeding positive lifestyle changes) (45, 60–62) as well as something that could be influenced by structural factors or external interventions (43, 56).

Six studies specifically examined how feelings of dismissal in interactions with health professionals influenced consumers' poor physical health care (28, 48, 63–66). This phenomenon was also referenced in passing in seven other studies (67–73). Across these studies, the researchers reported that consumers often experienced dismissal and invalidation of their physical health concerns by both mental health and physical health providers. While this can be understood as an effect of diagnostic overshadowing, there remains a knowledge gap regarding how the emotional experience of feeling dismissed influences help-seeking behaviors for both physical and mental health.

### 4.2. Social and structural aspects of physical health and mental health

In this section we consider how the social aspects of physical and mental health were reported in the literature. We review the research on consumers' experiences of stigma, how their wellbeing influences social participation, and the role of structural factors on their physical and mental health.

#### 4.2.1. Experiences of stigma within the health system

A total of 40 papers raised stigma as a factor in their findings. Fourteen articles reported that stigma, whether directly experienced or anticipated, negatively impacted consumers' physical health (53, 66, 69, 70, 72–81). Consumers experienced stigma, or an experience of judgement or devaluation, not only about mental health concerns but also about reproductive health and fertility (75), diabetes (72, 74), cardiovascular health (53), and other health behaviors (68, 76). A survey conducted by the Mental Health Council of Australia (73) found that mental health consumers experienced stigma in their mental health care and physical health care. Related research has found that consumers experience stigma in both primary care (79) and specialist care settings (66).

The literature itself reflects some of this stigma. For example, in Browne et al. (43), clinicians described consumers as largely inactive, while the consumers in that study identified that they did exercise, viewing it as important for their mental and physical health. In another example, Katakura et al. (82) describe a nurse using deceit and misinformation to trick a consumer into eating a different diet in order to manipulate health changes by telling her that certain recipes would remove her laugh lines and improve her aesthetic appearance.

#### 4.2.2. Social and interpersonal factors

Other social factors discussed included the impacts of physical and mental health concerns on a sense of self and social relationships. Social factors, such as social relationships and engaging in community support, were described as both enabling and hindering health behaviors. Multiple studies reported that these fears of stigma or discrimination, rather than symptomology, limited consumers' abilities to participate in social activities, including exercise programs (69, 83, 84). Additionally, social and cultural attitudes toward food and eating posed challenges to consumers trying to follow dietary recommendations (62, 85). Cabassa et al. (63) identified various social factors that consumers felt caused social friction when seeking treatment, including avoidance of disagreement, distrust, and differing cultural standards around body image. Furthermore, several studies dedicated to smoking-cessation interventions noted the social support required to cease smoking, with consumers suggesting that smoke-free social environments would best support them to quit (56, 80).

Social factors also informed how consumers conceptualized health more broadly. Consumers perceived healthy living to go beyond healthy eating and exercise to include friendship, secure and affordable housing, work, and spiritual and emotional health (69). Positive social support was considered by many to be an important facilitator of healthy lifestyle choices; conversely, a lack of social support was seen as a hindrance to adopting such behaviors (59, 86, 87).

#### 4.2.3. Structural factors that impact physical health and mental health

In 16 articles, consumers identified the inaccessibility of services as a significant barrier to their pursuit of physical health (43, 52, 71, 77, 82–84, 87–95). Several studies reported consumers' experiences of structural factors such as financial concerns and transport challenges as barriers to accessing necessary healthcare or influencing physical health decisions (43, 66, 77, 90). In particular, consumers felt their health and agency around healthcare was negatively impacted by the separation of mental and physical health care services and favored integrated care models with co-located services (52, 71, 83, 89, 92).

In two articles, consumers discussed how transport difficulties limited access to primary health providers and specialists (77, 88).^4^ However, another study comparing consumer and clinician perspectives found that transport was seen as a greater obstacle by clinicians than by consumers (43). Economic circumstances were widely identified by consumers as central to service accessibility (71, 77, 83, 88, 90, 93, 94) and ability to participate in lifestyle interventions addressing physical health concerns (84), a finding which was consistent across both geographical location and the timespan of our review. Vennedey et al. (95) describe the challenges consumers face navigating insurance schemes and other financial institutions. Finances also played a significant role in consumers' food choices and eating, with financial constraints affecting nutrition, food accessibility, and food security or insecurity, which had a bi-directional impact on health (62, 87).^5^ There was limited discussion of consumers' employment status; only one study reported consumers' desire to attain employment as a motivating factor toward health behaviors (82), and only four centered lack of employment as a contributing factor (41, 74, 78, 102). The financial constraints produced by unemployment, underemployment or insecure employment were largely unacknowledged.

### 4.3. Summary of findings

We found that the literature reviewed focused largely on consumers' individual choices and treatments, with significant attention on the physical impacts of neuroleptic medication and lifestyle changes that can improve poor health indicators, such as exercise, diet and smoking cessation. This pattern suggests that consumer-focused research into this subject perpetuates expectations that the burden of good mental health and physical wellbeing health largely falls on consumers and their individual choices. Discussions of how consumers' physical and mental health impacted their social relationships and civic participation further emphasized this individualistic focus. However, some of the literature captured consumers' understandings and reflections on how their physical and mental health interacts with systemic factors. The findings related to stigma, especially stigmatizing encounters within the healthcare system, and acknowledgment of structural factors informing health and health-seeking behaviors, such as transport, financial support, and accessibility of appropriate services, indicates that consumers' physical and mental health cannot be treated in a vacuum.

## 5. Challenges and gaps

In this section we summarize the challenges and gaps in the literature. The most critical challenge is the extent to which researchers filter consumer perspectives, a reflection of the lack of coproduced and codesigned research.

### 5.1. Filtered consumer perspectives

This review identified some issues with the way that non-consumer-identifying researchers framed consumer perspectives.^6^ In all research, prevailing orthodoxy and other factors contribute to the researcher's choice of research topic, research questions, framing, method, analysis, drafting choices, and conclusions. In mental health and other areas of research where existing power dynamics profoundly contribute to the phenomena being studied, it is vital that research does not mirror and reinforce such power asymmetries. This literature review revealed a consistent trend of not foregrounding consumer voices, or heavily filtering these voices *via* the researchers' perspectives. Of the 116 studies included in this review, 36% (*n* = 42) examined dual perspectives of consumers and clinicians and/or carers. Of these, four studies asked consumers only for their perspectives on the individual and/or personal factors influencing their health decisions (79, 90, 93, 103). Such research protocols over-emphasize consumers' individual agency (103), minimize consumer voices (104), or report clinician or carer views only on structural reforms required (90). One way to address this is to ensure consumer control of, and participation in, project design, development and implementation; very few studies taking this approach were present in the literature.

### 5.2. Lack of coproduced/codesigned projects

Only 15 studies had an identifiable lived experience element and only three (71, 74, 84) described the approach to codesign or coproduction.^7^ A further four used a participatory action research approach (54, 55, 105, 106). Ten studies noted that consumer researchers were members of the research team or conducted interviews (29, 36, 70, 71, 78, 84, 88, 107, 108).

This finding is significant, as participatory methods are considered the gold standard in health system research (109). While it is possible that some studies may have employed these methods but did not describe them, if this is the case, it nonetheless underscores the lack of importance accorded participatory methods. Either way, the fact that only a minority of the studies included such methods points to a significant knowledge gap.

## 6. Recommendations for future research

One of the key findings of this review is that research directly investigating consumers' experiences of systemic and structural factors which shape their physical and mental health care remains scant. Many of the studies included in this review focused solely on individual factors, such as motivation, side effects of medication, and the importance of social relationships to the health of consumers. These studies either did not ask participants about their experience with respect to the healthcare system, or only explored these issues with clinicians or carers.

When consumers are engaged in research to provide their perspective on factors that influence their health but are not invited to consider the structural and systemic factors, important information is missed. Such omissions reinforce the view that poor health is the individual's responsibility rather than a part and product of systemic factors, including the socio-cultural-political climate, race, ethnicity, disability and chronic health status, gender identity and expression, sexuality, refugee and migrant status, economic circumstances, education status, experience of incarceration, and geographical location. Future research, in alignment with the World Health Organization's (23) push to understand the social and structural factors that inform mental and physical health inequalities, should seek to redress this trend and ensure that consumers are given the opportunity to be included in these discussions and reflect upon and report their understandings of the full range of factors that they consider affect their health status.

Secondly, we have observed a clear gap between the research goals of the studies reviewed (to focus on the experiences of consumers) and their methods, which often implicitly appeared to devalue these experiences. Accordingly, we urge journals, ethics committees and research policy organizations to develop guidelines and standards to inform best practice in research on consumer perspectives and experience. Researchers are urged to implement codesigned and/or coproduced approaches to future study designs. There are examples of journals showing leadership by taking this stance and mandating standards with research involving disempowered populations, such as Indigenous peoples (110). Consistent with Lock et al. (110), it is clear that research in these contexts requires a different approach, necessitating additional resourcing, support and timelines. As noted above, the studies reviewed consistently followed widely accepted scholarly protocols for qualitative analysis. However, participatory methods that prioritize the sharing of power in the development of research foci, interview questions, interpreting results, and writing, lead to findings and recommendations that are more closely aligned with the concerns and perspectives of people who access mental health services (111). Coproduction and codesign are effective ways to center the health experiences of consumers and acknowledge and address existing power imbalances in partnerships between academic researchers and consumers (112). It is only through this paradigmatic shift that we can disrupt academic research's tendency to reproduce representations of mental health and physical health concerns as individual problems to be addressed by promoting help-seeking behaviors and lifestyle interventions. By centring consumer voices and needs, we can begin to address the complex challenges of physical health and mental health as they are experienced.

Health is a basic human right, yet the literature around consumer perspectives bears almost no discussion of how these rights might be actualized (113). As Daya et al. (114) argue, authentic engagement with lived experience requires listening to, and responding to, a diverse range of consumer experiences. This also results in better quality research (115). Through stronger and more equitable engagement with consumers, we can locate alternative pathways toward person-centered models of health and move toward policy change that might begin to redress the poorer health outcomes and reduced life expectancy of this group of people. While coproduction and/or codesign approaches are not a panacea and continue to suffer from a lack of clarity and definition (111, 116), further research into health-related outcomes must involve consumer voices as an integral part of the design of their research and interpretation of the results, and not just as research subjects. This can be achieved in a range of ways, clearly articulated in the mental health codesign literature, but require engagement with power dynamics (117), an understanding of the emotional labor required (118) and a reckoning of privilege within the institutions that conduct research and provide services (119).

## Author contributions

CG, CM, NH, KJ-A, RK, SDMT, and RR contributed to conception and design of the study. CG reviewed the literature and wrote the first draft of the manuscript. All authors contributed to manuscript revision, read, and approved the submitted version.

## Footnotes

^1^Our use of the term “mental health consumer” is guided in part by the language decided upon by our Consumer Leadership Advisory Group, and in part by the response from the Victorian Mental Illness Awareness Council Declaration, which found that 66% of participants who contributed to the Declaration tolerated this terminology (3). We understand that there are innumerable views on what terminology best represents the experiences of persons with mental health concerns, and our choice is intended to be non-pathologising and person-centred.

^2^Per Grant and Booth (33), a critical review involves the evaluation of the quality of a data set, and the interpretation of the data through analysis and synthesis of a diverse range of sources.

^3^Google Scholar is one option for conducting a gray literature search, which aims to capture non-published academic research (34). Our gray literature search strategy was developed in consultation with RMIT University's Library Services, and given the wide range of disciplines of the researchers involved in this study, it was decided that a Google Scholar search would capture a wide range of possible sources for inclusion, while not prohibiting research from certain areas from slipping through database cracks.

^4^Transport was noted in passing in a further 17 studies.

^5^This finding is consistent with literature findings that food insecurity has a significant impact on multiple domains of both mental and physical health, use of mental and physical health services, and medication use (96–101).

^6^We note that many researchers may have lived experience of mental health concerns, but that this does not make them consumer perspective researchers (30).

^7^We note Davidsen et al. (104) described their study as codesign; however, the method does not describe a codesign approach. This is likely to be a different use of the term codesign than is usually understood in mental health research contexts.

## References

[1] Roberts R Johnson C Hopwood M Firth J Jackson K Sara G. The potential impact of a public health approach to improving the physical health of people living with mental illness. Int J Environ Res Public Health. (2022) 19:11746. 10.3390/ijerph191811746 36142019 PMC9516962

[2] Roberts R Lockett H Bagnall C Maylea C Hopwood M. Improving the physical health of people living with mental illness in Australia and New Zealand. Aust J Rural Health. (2018) 26:354–62. 10.1111/ajr.12457 30303285

[3] VMIAC. VMIAC Declaration. (2019). Available online at: https://www.vmiac.org.au/declaration/ (accessed January 25, 2023).

[4] Daya I. The 50% challenge—Embracing the consumer workforce. In: New Paradigm. Spring (2015). p. 20–7.

[5] Roberts R. The Physical Health of People Living with a Mental Illness: A Narrative Literature Review. NSW: Charles Sturt University. (2019). Available online at: https://www.equallywell.org.au/wp-content/uploads/2019/10/Literature-review-EquallyWell-2a.pdf (accessed January 25, 2023).

[6] Erlangsen A Andersen PK Toender A Laursen TM Nordentoft M Canudas-Romo V. Cause-specific life-years lost in people with mental disorders: a nationwide, register-based cohort study. Lancet Psychiatry. (2017) 4:937–45. 10.1016/S2215-0366(17)30429-7 29122573

[7] Firth J Siddiqi N Koyanagi A Siskind D Rosenbaum S Galletly C. The Lancet Psychiatry Commission: A blueprint for protecting physical health in people with mental illness. Lancet Psychiatry. (2019) 6:675–712. 10.1016/S2215-0366(19)30387-6 31324560

[8] Lambert TJR Newcomer JW. Are the cardiometabolic complications of schizophrenia still neglected? Barriers to care. Med J Aust. (2009) 190:39. 10.5694/j.1326-5377.2009.tb02374.x 19220173

[9] Fagiolini A Frank E Scott JA Turkin S Kupfer DJ. Metabolic syndrome in bipolar disorder: Findings from the Bipolar Disorder Center for Pennsylvanians. Bipolar Disord. (2005) 7:424–30. 10.1111/j.1399-5618.2005.00234.x 16176435

[10] Lawrence D Hancock KJ Kisely S. The gap in life expectancy from preventable physical illness in psychiatric patients in Western Australia: Retrospective analysis of population based registers. BMJ. (2013) 346:f2539–f2539. 10.1136/bmj.f2539 23694688 PMC3660620

[11] Robson D Gray R. Serious mental illness and physical health problems: a discussion paper. Int J Nurs Stud. (2007) 44:457–66. 10.1016/j.ijnurstu.2006.07.013 17007859

[12] Australian Bureau of Statistics. Mortality of people using mental health services and prescription medications, analysis of 2011 data (4329.0.00.006). Australian Bureau of Statistics. (2017). Available online at: http://www.abs.gov.au/ausstats/abs@.nsf/0/EB5F81AAC6462C72CA2581B40012A37D?Opendocument (accessed January 25, 2023).

[13] Coghlan R Lawrence D Holman D Jablensky A. Duty to Care: Physical Illness in People with Mental Illness. Perth, WA: The University of Western Australia (2001).

[14] Druss BG Bradford DW Rosenheck RA Radford MJ Krumholz HM. Mental disorders and use of cardiovascular procedures after myocardial infarction. JAMA. (2000) 283:506. 10.1001/jama.283.4.506 10659877

[15] Lawrence DM Holman CDJ Jablensky AV Hobbs MST. Death rate from ischaemic heart disease in Western Australian psychiatric patients 1980–1998. Br J Psychiatry. (2003) 182:31–6. 10.1192/bjp.182.1.31 12509315

[16] Mitchell AJ Malone D Doebbeling CC. Quality of medical care for people with and without comorbid mental illness and substance misuse: systematic review of comparative studies. Br J Psychiatry. (2009) 194:491–9. 10.1192/bjp.bp.107.045732 19478286

[17] Hayes JF Marston L Walters K King MB Osborn DPJ. Mortality gap for people with bipolar disorder and schizophrenia: UK-based cohort study 2000–2014. Br J Psychiatry. (2017) 3:176–81. 10.1192/bjp.bp.117.202606 28684403 PMC5579328

[18] Hemingway H Marmot M. Psychosocial factors in the etiology and prognosis of coronary heart disease: Systematic review of prospective cohort studies. BMJ. (1999) 318:1460–7. 10.1136/bmj.318.7196.1460 10346775 PMC1115843

[19] Lawrence D Coghlan R. Health inequalities and the health needs of people with mental illness. N S W Public Health Bull. (2002) 13:155–8. 10.1071/NB02063 12451410

[20] Chen YH Lin HC Lin HC. Poor clinical outcomes among pneumonia patients with schizophrenia. Schizophr Bull. (2011) 37:1088–94. 10.1093/schbul/sbq019 20339152 PMC3160214

[21] Daumit GL Pronovost PJ Anthony CB Guallar E Steinwachs DM Ford DE. Adverse Events During Medical and Surgical Hospitalizations for Persons With Schizophrenia. Arch Gen Psychiatry. (2006) 63:267. 10.1001/archpsyc.63.3.267 16520431

[22] Kisely S Campbell LA Wang Y. Treatment of ischaemic heart disease and stroke in individuals with psychosis under universal healthcare. Br J Psychiatry. (2009) 195:545–50. 10.1192/bjp.bp.109.067082 19949207

[23] World Health Organization. Social Determinants of Mental Health. (2014). Available online at: https://www.who.int/publications/i/item/9789241506809 (accessed January 25, 2023).

[24] Molloy R Munro I Pope N. Understanding the experience of diagnostic overshadowing associated with severe mental illness from the consumer and health professional perspective: A qualitative systematic review protocol. JBI Evid Synth. (2020) 6:1362–8. 10.11124/JBIES-20-00244 33165171

[25] Kaine C Lawn S Roberts R Cobb L Erskine V. Review of Physical and Mental Health Care in Australia: A report by Lived Experience Australia Ltd and Equally Well. Lived Experience Australia Ltd, Equally Well (2022).

[26] Jones S Howard L Thornicroft G. ‘Diagnostic overshadowing': worse physical health care for people with mental illness. Acta Psychiatr Scand. (2008) 118:169–71. 10.1111/j.1600-0447.2008.01211.x 18699951

[27] Nash M. Diagnostic overshadowing: a potential barrier to physical health care for mental health service users. Ment Health Pract. (2013) 17:22–6. 10.7748/mhp2013.12.17.4.22.e862

[28] Happell B Ewart SB Platania-Phung C Bocking J Griffiths K Scholz B. Embedding a physical health nurse consultant within mental health services: consumers' perspectives. Int J Ment Health Nurs. (2016) 25:377–84. 10.1111/inm.12185 26748945

[29] Happell B Ewart SB Platania-Phung C Stanton R. Participative mental health consumer research for improving physical health care: an integrative review. Int J Ment Health Nurs. (2016) 25:399–408. 10.1111/inm.12226 27159221

[30] Happell B Roper C. Consumer participation in mental health research: articulating a model to guide practice. Aust Psychiatry. (2007) 15:237–41. 10.1080/10398560701320113 17516188

[31] World Health Organization. Comprehensive Mental Health Action Plan 2013–2030. World Health Organization. (2021). Available online at: https://www.who.int/publications/i/item/9789241506021 (accessed January 25, 2023).

[32] State of Victoria. Royal Commission into Victoria's Mental Health System. Victorian Government. (2021). Available online at: https://finalreport.rcvmhs.vic.gov.au/ (accessed January 25, 2023).

[33] Grant MJ Booth A. A typology of reviews: an analysis of 14 review types and associated methodologies. Health Info Libr J. (2009) 26:91–108. 10.1111/j.1471-1842.2009.00848.x 19490148

[34] Mahood Q Van Eerd D Irvin E. Searching for grey literature for systematic reviews: Challenges and benefits. Res Synth Methods. (2014) 5:221–34. 10.1002/jrsm.1106 26052848

[35] Blanner Kristiansen C Juel A Vinther Hansen M Hansen AM Kilian R Hjorth P. Promoting physical health in severe mental illness: patient and staff perspective. Acta Psychiatr Scand. (2015) 132:470–8. 10.1111/acps.12520 26696384

[36] Happell B Ewart SB Platania-Phung C Bocking J Scholz B Stanton R. What physical health means to me: perspectives of people with mental illness. Issues Ment Health Nurs. (2016) 37:934–41. 10.1080/01612840.2016.1226999 27786585

[37] Hemmings L Soundy A. experiences of physiotherapy in mental health: an interpretative phenomenological analysis of barriers and facilitators to care. Physiotherapy. (2020) 109:94–101. 10.1016/j.physio.2020.01.001 32522361

[38] Çelik Ince S Partlak Günüşen N Serçe Ö. Perception of physical health by patients with severe mental illness and their family caregivers: a qualitative study. Perspect Psychiatr Care. (2019) 55:718–27. 10.1111/ppc.12416 31292971

[39] Cullen C McCann E. Exploring the role of physical activity for people diagnosed with serious mental illness in Ireland: physical activity and people with SMI. J Psychiatr Ment Health Nurs. (2015) 22:58–64. 10.1111/jpm.12179 25490992

[40] Juel A Hjorth P Munk-Jørgensen P Buus N. Health beliefs and experiences of a health promotion intervention among psychiatric patients with substance use: an interview study. Arch Psychiatric Nurs. (2018) 32:462–8. 10.1016/j.apnu.2018.01.004 29784231

[41] Cimo A Dewa CS. Symptoms of mental illness and their impact on managing type 2 diabetes in adults. Can J Diabetes. (2018) 42:372–81. 10.1016/j.jcjd.2017.08.256 29128304

[42] Morant N Azam K Johnson S Moncrieff J. The least worst option: User experiences of antipsychotic medication and lack of involvement in medication decisions in a UK community sample. J Ment Health. (2018) 27:322–8. 10.1080/09638237.2017.1370637 28857636

[43] Browne J Mihas P Penn DL. Focus on exercise: client and clinician perspectives on exercise in individuals with serious mental illness. Community Ment Health J. (2016) 52:387–94. 10.1007/s10597-015-9896-y 26007648

[44] Patel P Frederick T Kidd SA. Physical health, community participation and schizophrenia. J Health Psychol. (2018) 23:79–83. 10.1177/1359105316666654 27624616

[45] Abed H. What factors affect the lifestyle choices of people with schizophrenia? Ment Health Rev J. (2010) 15:21–7. 10.5042/mhrj.2010.0368

[46] Cocoman AM Casey M. The physical health of individuals receiving antipsychotic medication: a qualitative inquiry on experiences and needs. Issues Ment Health Nurs. (2018) 39:282–9. 10.1080/01612840.2017.1386744 29333898

[47] McAulay C Dawson L Mond J Outhred T Touyz S. “The Food Matches the Mood”: experiences of eating disorders in bipolar disorder. Qual Health Res. (2021) 31:100–12. 10.1177/1049732320956267 32940133

[48] Woods T. Physical health care challenges for consumers of mental health services. Psychiatr Rehabil J. (2011) 34:332–3. 10.2975/34.4.2011.332.333 21459752

[49] Young SJ Praskova A Hayward N Patterson S. Attending to physical health in mental health services in Australia: a qualitative study of service users' experiences and expectations. Health Soc Care Community. (2017) 25:602–11. 10.1111/hsc.12349 27093882

[50] Nakanishi M Tanaka S Kurokawa G Ando S Yamasaki S Fukuda M. Inhibited autonomy for promoting physical health: Qualitative analysis of narratives from persons living with severe mental illness. BJPsych Open. (2019) 5:e10. 10.1192/bjo.2018.77 30762505 PMC6343122

[51] Kreyenbuhl J Lucksted A Despeaux K Sykes VM. Understanding women veterans' experiences with and management of weight gain from medications for serious mental illness: a qualitative study. Psychiatr Rehabil J. (2019) 42:238–45. 10.1037/prj0000348 30920258 PMC11825350

[52] Rollins AL Wright-Berryman J Henry NH Quash AM Benbow K Bonfils KA. Managing physical and mental health conditions: Consumer perspectives on integrated care. Soc Work Ment Health. (2017) 15:66–79. 10.1080/15332985.2016.1173160 29308057 PMC5754195

[53] Ralat SI Depp CA Bernal G. Reasons for nonadherence to psychiatric medication and cardiovascular risk factors treatment among latino bipolar disorder patients living in Puerto Rico: a qualitative study. Community Ment Health J. (2018) 54:707–16. 10.1007/s10597-017-0202-z 29127563 PMC5945339

[54] Ross LE Vigod S Wishart J Waese M Spence JD Oliver J. Barriers and facilitators to primary care for people with mental health and/or substance use issues: a qualitative study. BMC Fam Pract. (2015) 16:135. 10.1186/s12875-015-0353-3 26463083 PMC4604001

[55] Werremeyer A Skoy E Aalgaard Kelly G. Use of photovoice to understand the experience of taking psychotropic medications. Qual Health Res. (2017) 27:1959–69. 10.1177/1049732317693221 29088990

[56] Rae J Pettey D Aubry T Stol J. Factors affecting smoking cessation efforts of people with severe mental illness: a qualitative study. J Dual Diagn. (2015) 11:42–49. 10.1080/15504263.2014.992096 25491704

[57] Kerr S Woods C Knussen C Watson H Hunter R. Breaking the habit: a qualitative exploration of barriers and facilitators to smoking cessation in people with enduring mental health problems. BMC Public Health. (2013) 13:221. 10.1186/1471-2458-13-221 23497231 PMC3599988

[58] Bochicchio L Stefancic A Gurdak K Swarbrick M Cabassa LJ. “We're All in this Together”: peer-specialist contributions to a healthy lifestyle intervention for people with serious mental illness. Adm Policy Ment Health. (2019) 46:298–310. 10.1007/s10488-018-0914-6 30565004 PMC6459695

[59] Graham C Rollings C de Leeuw S Anderson L Griffiths B Long N. A qualitative study exploring facilitators for improved health behaviors and health behavior programs: mental health service users' perspectives. Sci World J. (2014) 2014:1–7. 10.1155/2014/870497 24895667 PMC4033592

[60] Hassan S Ross J Marston L Burton A Osborn D Walters K. Exploring how health behaviours are supported and changed in people with severe mental illness: a qualitative study of a cardiovascular risk reducing intervention in Primary Care in England. Br J Health Psychol. (2020) 25:428–51. 10.1111/bjhp.12415 32281720

[61] Gandhi S Gurusamy J Damodharan D Ganesan V Palaniappan M. Facilitators of healthy life style behaviors in persons with schizophrenia—A qualitative feasibility pilot study. Asian J Psychiatr. (2019) 40:3–8. 10.1016/j.ajp.2019.01.003 30658242

[62] Sayer J Paniagua D Ballentine S Sheehan L Carson M Nieweglowski K. Perspectives on diet and physical activity among urban African Americans with serious mental illness. Soc Work Health Care. (2019) 58:509–25. 10.1080/00981389.2019.1587662 30907271 PMC6658098

[63] Cabassa LJ Siantz E Nicasio A Guarnaccia P Lewis-Fernández R. Contextual factors in the health of people with serious mental illness. Qual Health Res. (2014) 24:1126–37. 10.1177/1049732314541681 24966198 PMC4276729

[64] Ewart SB Bocking J Happell B Platania-Phung C Stanton R. Mental health consumer experiences and strategies when seeking physical health care: a focus group study. Glob Qual Nurs Res. (2016) 3:233339361663167. 10.1177/2333393616631679 28462330 PMC5342294

[65] Jerwood J Ward G Phimister D Holliday N Coad J. Lean in, don't step back: The views and experiences of patients and carers with severe mental illness and incurable physical conditions on palliative and end of life care. Prog Palliat Care. (2021) 5:1–9. 10.1080/09699260.2021.1887589

[66] Nash M. Mental health service users' experiences of diabetes care by Mental Health Nurses: An exploratory study: physical health and mental health. J Psychiatr Ment Health Nurs. (2014) 21:715–23. 10.1111/jpm.12140 24548452

[67] Burton A Osborn D Atkins L Michie S Gray B Stevenson F. Lowering cardiovascular disease risk for people with severe mental illnesses in primary care: a focus group study. PLoS ONE. (2015) 10:e0136603. 10.1371/journal.pone.0136603 26317516 PMC4552729

[68] Ehrlich C Chester P Kisely S Crompton D Kendall E. Making sense of self-care practices at the intersection of severe mental illness and physical health—An Australian study. Health Soc Care Community. (2018) 26:e47–55. 10.1111/hsc.12473 28685496

[69] Graham C Griffiths B Tillotson S Rollings C. Healthy living? By whose standards? Engaging mental health service recipients to understand their perspectives of, and barriers to, healthy living. Psychiatr Rehabil J. (2013) 36:215–8. 10.1037/prj0000009 23876179

[70] Hamilton S Pinfold V Cotney J Couperthwaite L Matthews J Barret K. Qualitative analysis of mental health service users' reported experiences of discrimination. Acta Psychiatr Scand. (2016) 134:14–22. 10.1111/acps.12611 27426642 PMC6680261

[71] Happell B Platania-Phung C Bocking J Ewart SB Scholz B Stanton R. Consumers at the centre: Interprofessional solutions for meeting mental health consumers' physical health needs. J Interprof Care. (2019) 33:226–34. 10.1080/13561820.2018.1516201 30257120

[72] Knyahnytska Y Williams C Dale C Webster F. Changing the conversation: diabetes management in adults with severe mental illnesses and type 2 diabetes. Can J Diabetes. (2018) 42:595–602. 10.1016/j.jcjd.2018.02.001 29861331

[73] Mental Health Council of Australia. Consumer and Carer Experiences of Stigma from Mental Health and Other Health Professionals. Canberra, ACT: Mental Health Council of Australia (2011).

[74] Bellass S Lister J Kitchen CEW Kramer L Alderson SL Doran T. Living with diabetes alongside a severe mental illness: a qualitative exploration with people with severe mental illness, family members and healthcare staff Diabetic Medicine. J Br Diabetic Assoc. (2021) 38:e14562. 10.1111/dme.14562 33772867

[75] Birch S Lavender T Cupitt C. The physical healthcare experiences of women with mental health problems: status versus stigma. J Ment Health. (2005) 14:61–72. 10.1080/09638230500048032

[76] Björk Brämberg E Torgerson J Norman Kjellström A Welin P Rusner M. Access to primary and specialized somatic health care for persons with severe mental illness: a qualitative study of perceived barriers and facilitators in Swedish health care. BMC Fam Pract. (2018) 19:12. 10.1186/s12875-017-0687-0 29316894 PMC5759233

[77] Blixen CE Kanuch S Perzynski AT Thomas C Dawson NV Sajatovic M. Barriers to self-management of serious mental illness and diabetes. Am J Health Behav. (2016) 40:194–204. 10.5993/AJHB.40.2.4 26931751 PMC4928189

[78] Lakeman R McGowan P MacGabhann L Parkinson M Redmond M Sibitz I. A qualitative study exploring experiences of discrimination associated with mental-health problems in Ireland. Epidemiol Psychiatr Sci. (2012) 21:271–9. 10.1017/S2045796012000017 22794274

[79] McCabe MP Leas L. A qualitative study of primary health care access, barriers and satisfaction among people with mental illness. Psychol Health Med. (2008) 13:303–12. 10.1080/13548500701473952 18569898

[80] Okoli CTC El-Mallakh P Seng S. Which types of tobacco treatment interventions work for people with schizophrenia? Provider and mental health consumer perspectives. Issues Ment Health Nurs. (2019) 40:870–9. 10.1080/01612840.2018.1490833 30388915

[81] Stanhope V Henwood BF. Activating people to address their health care needs: learning from people with lived experience of chronic illnesses. Community Ment Health J. (2014) 50:656–63. 10.1007/s10597-013-9686-3 24337522

[82] Katakura N Matsuzawa K Ishizawa K Takayanagi C. Psychological and physical self-management of people with schizophrenia in community psychiatric rehabilitation settings: a qualitative study: health self-management in psychosis. Int J Nurs Pract. (2013) 19:24–33. 10.1111/ijn.12041 23617446

[83] Kemp V Fisher C Lawn S Battersby M Isaac MK. Small steps: Barriers and facilitators to physical health self-management by people living with mental illness. Int J Ment Health Promot. (2014) 16:216–30. 10.1080/14623730.2014.931069

[84] Wheeler AJ Roennfeldt H Slattery M Krinks R Stewart V. Codesigned recommendations for increasing engagement in structured physical activity for people with serious mental health problems in Australia. Health Soc Care Community. (2018) 26:860–70. 10.1111/hsc.12597 30047608

[85] Barre LK Ferron JC Davis KE Whitley R. Healthy eating in persons with serious mental illnesses: understanding and barriers. Psychiatr Rehabil J. (2011) 34:304–10. 10.2975/34.4.2011.304.310 21459746

[86] Hultsj ö S Blomqvist KB. Health behaviors as conceptualised by individuals diagnosed with a psychotic disorder. Issues Ment Health Nurs. (2013) 34: 665–672. 10.3109/01612840.2013.794178 24004360

[87] Jimenez DE Aschbrenner K Burrows K Pratt SI Alegría M Bartels SJ. Perspectives of overweight Latinos with serious mental illness on barriers and facilitators to health behavior change. J Lat Psychol. (2015) 3:11–22. 10.1037/lat0000020 25664227 PMC4314710

[88] Ewart SB Happell B Bocking J Platania-Phung C Stanton R Scholz B. Social and material aspects of life and their impact on the physical health of people diagnosed with mental illness. Health Expect. (2017) 20:984–91. 10.1111/hex.12539 28295883 PMC5600237

[89] Knowles SE Chew-Graham C Adeyemi I Coupe N Coventry PA. Managing depression in people with multimorbidity: a qualitative evaluation of an integrated collaborative care model. BMC Fam Pract. (2015) 16:32. 10.1186/s12875-015-0246-5 25886864 PMC4355419

[90] Lee S Waters F Briffa K Fary RE. Limited interface between physiotherapy primary care and people with severe mental illness: a qualitative study. J Physiother. (2017) 63:168–74. 10.1016/j.jphys.2017.05.014 28652081

[91] Melamed OC Fernando I Soklaridis S Hahn MK LeMessurier KW Taylor VH. Understanding engagement with a physical health service: a qualitative study of patients with severe mental illness. Can J Psychiatry. (2019) 64:872–80. 10.1177/0706743719862980 31303027 PMC7003111

[92] Mikocka-Walus A Hanlon I Dober M Emerson C Beswick L Selinger C. Lived experience in people with inflammatory bowel disease and comorbid anxiety and depression in the United Kingdom and Australia. J Health Psychol. (2020) 12:2290–303. 10.1177/1359105320911427 32175775

[93] Roberge P Hudon C Pavilanis A Beaulieu M-C Benoit A Brouillet H. A qualitative study of perceived needs and factors associated with the quality of care for common mental disorders in patients with chronic diseases: The perspective of primary care clinicians and patients. BMC Fam Pract. (2016) 17:134. 10.1186/s12875-016-0531-y 27620166 PMC5020556

[94] Schmutte T Flanagan E Bedregal L Ridgway P Sells D Styron T. Self-efficacy and self-care: missing ingredients in health and healthcare among adults with serious mental illnesses. Psychiatr Q. (2009) 80:1–8. 10.1007/s11126-008-9088-9 19048375

[95] Vennedey V Hower KI Hillen H Ansmann L Kuntz L Stock S. Patients' perspectives of facilitators and barriers to patient-centred care: Insights from qualitative patient interviews. BMJ Open. (2020) 10:e033449. 10.1136/bmjopen-2019-033449 32376748 PMC7223019

[96] Afulani PA Coleman-Jensen A Herman D. Food insecurity, mental health, and use of mental health services among nonelderly adults in the United States. J Hunger Environ Nutr. (2020) 15:29–50. 10.1080/19320248.2018.1537868

[97] Burruss NC Girgis M Green KE Lu L Palakshappa D. Association between food insecurity and access to a mental health professional: Cross-sectional analysis of NHANES 2007–2014. BMC Public Health. (2021) 21:754. 10.1186/s12889-021-10818-5 33874932 PMC8054684

[98] Douglas F MacIver E Yuill C. A qualitative investigation of lived experiences of long-term health condition management with people who are food insecure. BMC Public Health. (2020) 20:1309. 10.1186/s12889-020-09299-9 32859179 PMC7456079

[99] Elgar FJ Pickett W Pförtner TK Gariépy G Gordon D Georgiades K. Relative food insecurity, mental health and wellbeing in 160 countries. Soc Sci Med. (2021) 268:113556. 10.1016/j.socscimed.2020.113556 33293171

[100] Jia J Fung V Meigs JB Thorndike AN. Food insecurity, dietary quality, and health care utilization in lower-income adults: a cross-sectional study. J Acad Nutr Dietetics. (2021) 121:2177–86.e3. 10.1016/j.jand.2021.06.001 34247978

[101] Whittle HJ Wolfe WR Sheira LA Frongillo EA Palar K Merenstein D. Associations between food insecurity and psychotropic medication use among women living with HIV in the United States. Epidemiol Psychiatr Sci. (2020) 29:e113. 10.1017/S2045796020000232 32248873 PMC7214522

[102] Carson NJ Katz AM Alegría M. How patients and clinicians make meaning of physical suffering in mental health evaluations. Transcult Psychiatry. (2016) 53:595–611. 10.1177/1363461516660901 27460985 PMC8043772

[103] Mc Sharry J Bishop FL Moss-Morris R Kendrick T. ‘The chicken and egg thing': Cognitive representations and self-management of multimorbidity in people with diabetes and depression. Psychol Health. (2013) 28:103–19. 10.1080/08870446.2012.716438 22924481

[104] Davidsen AS Davidsen J Jønsson ABR Nielsen MH Kjellberg PK Reventlow S. Experiences of barriers to trans-sectoral treatment of patients with severe mental illness. A qualitative study. Int J Ment Health Syst. (2020) 14:87. 10.1186/s13033-020-00419-x 33292415 PMC7706214

[105] Cabassa LJ Parcesepe A Nicasio A Baxter E Tsemberis S Lewis-Fernández R. Health and wellness photovoice project: engaging consumers with serious mental illness in health care interventions. Qual Health Res. (2013) 23:618–30. 10.1177/1049732312470872 23258117 PMC3818106

[106] Xiong G Ziegahn L Schuyler B Rowlett A Cassady D. Improving dietary and physical activity practices in group homes serving residents with severe mental illness. Prog Community Health Partnersh Res Educ Action. (2010) 4:279–88. 10.1353/cpr.2010.0015 21169705 PMC4412593

[107] Bocking J Ewart SB Happell B Platania-Phung C Stanton R Scholz B. “Here if you need me”: Exploring peer support to enhance access to physical health care. J Ment Health. (2018) 27:329–35. 10.1080/09638237.2017.1385741 29029587

[108] Happell B Ewart SB Bocking J Platania-Phung C Stanton R. ‘That red flag on your file': Misinterpreting physical symptoms as mental illness. J Clin Nurs. (2016) 25:2933–42. 10.1111/jocn.13355 27230306

[109] World Health Organization. World Report on Knowledge for Better Health: Strengthening Health Systems. World Health Organization. (2004). Available online at: https://apps.who.int/iris/handle/10665/43058 (accessed January 25, 2023).

[110] Lock M McMillan F Bennett B Martire JL Warne D Kidd J. Position statement: Research and reconciliation with Indigenous peoples in rural health journals. Aust J Rural Health. (2022) 30:6–7. 10.1111/ajr.12834 35043514

[111] Redman S Greenhalgh T Adedokun L Staniszewska S Denegri S. Coproduction of knowledge: The future. BMJ. (2021) 372:n434. 10.1136/bmj.n434 33593753 PMC7884902

[112] McMullin C Needham C. Coproduction in healthcare. In: Coproduction and Co-Creation: Engaging Citizens in Public Services. New York, NY: Routledge (2018). p. 151–60.

[113] Roberts R. Equally Well Consensus Statement. Improving the Physical Health Wellbeing of People Living with Mental Illness in Australia. Sydney, National Mental Health Commission. (2016). Available online at: https://www.equallywell.org.au/wp-content/uploads/2018/12/Equally-Well-National-Consensus-Booklet-47537.pdf (accessed January 25, 2023).

[114] Daya I Hamilton B Roper C. Authentic engagement: a conceptual model for welcoming diverse and challenging consumer and survivor views in mental health research, policy, and practice. Int J Ment Health Nurs. (2020) 29:299–311. 10.1111/inm.12653 31538723 PMC7328715

[115] Happell B Gordon S Bocking J Ellis P Roper C Liggins J. How did I not see that? Perspectives of nonconsumer mental health researchers on the benefits of collaborative research with consumers. Int J Ment Health Nurs. (2018) 27:1230–9. 10.1111/inm.12453 29527786

[116] Williams O Sarre S Papoulias SC Knowles S Robert G Beresford P. Lost in the shadows: Reflections on the dark side of coproduction. Health Res Policy Syst. (2020) 18:43. 10.1186/s12961-020-00558-0 32380998 PMC7204208

[117] Farr M. Power dynamics and collaborative mechanisms in co-production and co-design processes. Crit Soc Policy. (2018) 38:623–44. 10.1177/0261018317747444

[118] Faulkner A Thompson R. Uncovering the emotional labour of involvement and co-production in mental health research. Disabil Soc. (2021) 36:1–24. 10.1080/09687599.2021.1930519

[119] Rose D Kalathil J. Power privilege and knowledge: The untenable promise of co-production in mental “health”. Front Sociol. (2019) 4:57. 10.3389/fsoc.2019.00057 33869380 PMC8022626

